# Checklists of Parasites of Farm Fishes of Babylon Province, Iraq

**DOI:** 10.1155/2016/7170534

**Published:** 2016-07-31

**Authors:** Furhan T. Mhaisen, Abdul-Razzak L. Al-Rubaie

**Affiliations:** ^1^Tegnervägen 6B, 641 36 Katrineholm, Sweden; ^2^Department of Biological Control Technology, Al-Musaib Technical College, Al-Furat Al-Awsat Technical University, Al-Musaib, Iraq

## Abstract

Literature reviews of all references concerning the parasitic fauna of fishes in fish farms of Babylon province, middle of Iraq, showed that a total of 92 valid parasite species are so far known from the common carp (*Cyprinus carpio*), the grass carp (*Ctenopharyngodon idella*), and the silver carp (*Hypophthalmichthys molitrix*) as well as from three freshwater fish species (*Carassius auratus*,* Liza abu*, and* Heteropneustes fossilis*) which were found in some fish farms of the same province. The parasitic fauna included one mastigophoran, three apicomplexans, 13 ciliophorans, five myxozoans, five trematodes, 45 monogeneans, five cestodes, three nematodes, two acanthocephalans, nine arthropods, and one mollusc. The common carp was found to harbour 81 species of parasites, the grass carp 30 species, the silver carp 28 species,* L. abu* 13 species,* C. auratus* one species, and* H. fossilis* one species. A host-parasite list for each fish species was also provided.

## 1. Introduction

Although fish farming in Iraq started in 1955 with a small pond in Al-Zaafaraniya, south of Baghdad city [1], an advance was achieved in fish farming industry in Iraq during the seventies and early eighties of the last century when many fish farms were established especially in the middle of Iraq [2]. However, such achievement was hindered due to consequences of the war situations during 1980–1988 and 1991 as well as the economic sanction imposed by the UN against Iraq on August 6, 1990. During the last few years, a great advance was achieved in fish farming in general and fish cages in particular due to the increasing demand on fish protein as well as the increasing investment in fish-culture industry in most provinces of Iraq. According to the statistics, a total of 441 working fish farms are scattered in Iraq [3]. Of these farms, a total of 72 working fish farms are situated in Babylon province alone with a water area of 44.5% of the total water area of fish farms in Iraq.

Under extensive fish culture and inadequate administrative and control measures, fish farms are vulnerable to great hazards due to the infection with parasites and other disease agents [2, 4, 5]. Many parasite species can easily spread among fishes suffering from crowd and bad managements, especially those parasites with direct life cycles [6].

In connection with the parasites of cultured fishes of Babylon province, Mhaisen et al. [7] surveyed the literature on the parasitic fauna of fishes of Al-Furat Fish Farm (previously known as Babylon Fish Farm), which is the biggest fish farm in Babylon province, and showed that the parasitic fauna of fishes of that farm included 60 valid parasite species (10 protozoans, three myxozoans, one trematode, 29 monogeneans, five cestodes, three nematodes, two acanthocephalans, six crustaceans, and one mollusc larva). In addition, some investigations on other fish farms in Babylon province were done. Such data are scattered in different local journals, unpublished theses, and few other sources. Therefore, the present paper was aimed at gathering data from the literature concerning all fish farms of Babylon province and providing a list of parasite species according to their major groups as well as a host-parasite list for cultured fishes of these farms and some other fish species found in such farms. Such parasite list will help owners of fish farms and fish veterinarians to know what sort of parasites are found in their fish farms, which will help them later in taking appropriate measures for their control.

## 2. Sources and Methods

A total of 50 references (33 published articles, 12 unpublished theses, two unpublished reports, one book, one conference abstract, and one review article) dealing with the parasites of farm fishes of Babylon province were used to prepare the present paper. Data from such references was gathered to provide host-parasite and parasite-host lists. The systematic account of these parasites is based on some electronic sites [8–12] as well as some taxonomic references [13–16].

The index-catalogue of parasites and disease agents of fishes of Iraq [17] was used to indicate the total number of fish hosts harbouring each parasite species in the whole waters of Iraq.

## 3. Parasitological Investigations Achieved on Fish Farms of Babylon Province

Few fish farms in Babylon province were surveyed for some parasitic infections. Fishes from Al-Furat Fish Farm received the greatest attention in this respect. So far 25 chronologically arranged references [18–42] were concerned with the parasitic fauna of Al-Furat Fish Farm. Only seven references [43–49] were concerned with the parasitic fauna of Al-Shark Al-Awsat Fish Farm. The literature concerned with fish parasites of other farms in Babylon province included those from Al-Latifiya Fish Farm [6, 50–52]; Al-Bajaa Fish Farm [26]; Abdul-Razzak Al-Janabi Fish Farm; Fawzi Al-Janabi Fish Farm and Ali Al-Hayali Fish Farm [53]; three fish farms at Al-Iskandariya district: Abdul-Hadi Al-Matloob Fish Farm, Hussain Al-Gaiem Fish Farm, and Maki Chinak Fish Farm [54]; Technical Institute of Al-Musaib Fish Farm [55]; and Al-Manahil (Al-Bilad) Fish Farm at Al-Iskandariya district [56]. In addition, surveys were done from some unnamed fish ponds such as those at Al-Mahaweel district [57], Al-Musaib district [58], Al-Iskandariya district [59], and Sadat Al-Hindiya district [60] as well as some other unnamed farms in the province [61–65].

## 4. Results and Discussion

Surveying the literature concerning the parasites so far recorded from fish farms of Babylon province showed the presence of 92 parasite species. These parasites included one mastigophoran, three apicomplexans, 13 ciliophorans, five myxozoans, five trematodes, 45 monogeneans, five cestodes, three nematodes, two acanthocephalans, nine arthropods, and one mollusc. The common carp was found to harbour 81 species of parasites, the grass carp 30 species, the silver carp 28 species,* L. abu* 13 species,* C. auratus* one species, and* H. fossilis* one species. The layout and names of the major taxonomic groups (phyla and classes) followed a checklist of an FAO Fisheries Technical Paper [66]. These major groups represent the concerned phyla of the parasites, but due to the great numbers of parasite species of the phylum Platyhelminthes, its three classes (Trematoda, Monogenea, and Cestoda) were applied in addition to their phylum.

## 5. Major Groups of Parasitic Fauna: Parasite-Host List

The parasite-host list is arranged in the major groups (phyla or classes) of parasitic fauna according to Kirjušina and Vismanis [66]. For each major group, a list of species together with their hosts and concerned references is given. To economize space, names of fish farms are not given here as they can be easily detected from the previous subtitle “Parasitological Investigations Achieved on Fish Farms of Babylon Province.” Also the systematic account of all major groups is given down to the specific name of all parasites. For each parasite species, all records in farm fishes in Babylon province are given together with the first record of each concerned parasite in Iraq as well as the present number of all hosts so far known in Iraq for each concerned species based on the index-catalogue of parasites and disease agents of fishes of Iraq [17].

### 5.1. Phylum Mastigophora

The phylum Mastigophora is represented in farm fishes of Babylon province with only one parasite species of the genus* Ichthyobodo*. The systematic account of this parasite, followed by parasite-host list, is given here.

 Phylum Mastigophora

 Class Kinetoplastidea
   Order Kinetoplastida
     Family Bodonidae
       
*Ichthyobodo necator* (Henneguy, 1884) Pinto, 1928

*Ichthyobodo necator* (Henneguy, 1884) Pinto, 1928, was erroneously reported as* Costia necatrix* from the skin and gills of* C. carpio* [27]. The first record of* C. necatrix* in Iraq was from body surface of* H. fossilis* from Al-Ashar Canal at Basrah [67]. Seven fish host species are so far known for this parasite (as* C. necatrix*) in Iraq [17].

### 5.2. Phylum Apicomplexa

The phylum Apicomplexa, which is known as phylum Myzozoa according to WoRMS [12], is represented in farm fishes of Babylon province with three species; two of them belonged to the genus* Eimeria* and one unspecified species to the genus* Haemogregarina*. 

 Phylum Apicomplexa

 Class Sporozoa
   Order Eucoccidiorida
     Family Eimeriidae
       
*Eimeria dogieli* (Dogiel, 1948) Pellerdy, 1963
       
*Eimeria mylopharyngodoni* Chen, 1956
     Family Haemogregarinidae
       
*Haemogregarina* sp.

*Eimeria dogieli* (Dogiel, 1948) Pellerdy, 1963, was recorded from the intestine of* C. carpio* [27]. So far, this is the only record of* E. dogieli* from fishes of Iraq [17].

*Eimeria mylopharyngodoni* Chen, 1956, was recorded from* C. carpio* [49]. The specific name was misspelled as* mylopharyngodon* and no authority, site of infection, parasite description, and illustration were given for this parasite by Hussain et al. [49]. So this record is considered as questionable especially if we take in consideration that Hussain et al. [49] examined* C. carpio* externally while* E. mylopharyngodoni* is known to infect intestine, kidneys, and liver of fishes [68]. This is the only record of* E. mylopharyngodoni* from fishes of Iraq [17].

*Haemogregarina* sp. was found on gills of* C. carpio* [49]. In Iraq, three species of* Haemogregarina* were so far recorded from blood of three fish species in Basrah province only [17]. So we think that the record of* Haemogregarina* sp. from gills of* C. carpio* by Hussain et al. [49] with neither description nor a good illustration is considered as questionable.

### 5.3. Phylum Ciliophora

The phylum Ciliophora is represented in farm fishes of Babylon province with 13 species, three of which belonged to the genera* Chilodonella*,* Ichthyophthirius*, and* Tripartiella*, five to the genus* Apiosoma*, and four to the genus* Trichodina* in addition to unspecified species of the genus* Trichodina*. 

 Phylum Ciliophora

 Class Kinetophragminophorea
   Order Cyrtophorida
     Family Chilodonellidae
       
*Chilodonella cyprini *(Moroff, 1902) Strand, 1928
 Class Oligohymenophorea
   Order Hymenostomatida
     Family Ichthyophthiriidae
       
*Ichthyophthirius multifiliis* Fouquet, 1876
   Order Petrichida
     Family Epistylididae
       
*Apiosoma amoebae* (Grenfell, 1887) Lom, 1966
       
*Apiosoma cylindriformis* (Chen, 1955)
       
*Apiosoma minuta* (Chen, 1961) Lom, 1966
       
*Apiosoma piscicola* Blanchard, 1885
       
*Apiosoma poteriformis* (Timofeev, 1962) Lom, 1966
   Order Mobilida
     Family Trichodinidae
       
*Trichodina cottidarum* Dogiel, 1948
       
*Trichodina domerguei* (Wallengren, 1897)
       
*Trichodina gracilis* Polyanskii, 1955
       
*Trichodina nigra* Lom, 1960
       
*Trichodina* sp.
       
*Tripartiella amurensis* (Chan, 1961)

*Chilodonella cyprini* (Moroff, 1902) Strand, 1928, was recorded from skin, buccal cavity, and gills of* C. idella* [27, 29, 43, 51], skin, fins, buccal cavity, and gills of* C. carpio* [27, 30, 43, 51, 53], and skin and gills of* H. molitrix* [27, 43, 51]. The first record of* C. cyprini* in Iraq was from skin, buccal cavity, and gills of* Mystus pelusius* from Tigris River at Baghdad [69]. So far 13 fish host species are known for this parasite in Iraq [17].

*Ichthyophthirius multifiliis* Fouquet, 1876, was recorded from skin and gills of* C. idella* [27, 29, 43, 51, 56], skin, fins, and gills of* C. carpio* [25, 27, 29, 30, 35, 43, 45, 47, 49, 51, 53–56, 59, 60, 65], and skin and gills of* H. molitrix* [27, 29, 45, 47, 51]. The first record of* I. multifiliis* in Iraq was from the skin and gills of* Chelon subviridis* (reported as* Mugil dussumieri*) from Tigris River near Baghdad [70]. So far this parasite has 35 fish host species in Iraq [17].

*Apiosoma amoebae* (Grenfell, 1887) Lom, 1966, was reported from skin, buccal cavity, and gills of* C. idella* [20, 24, 27], skin and buccal cavity of* C. carpio* [27, 55], and gills and buccal cavity of* H. molitrix* [20, 27]. It is appropriate to mention here that* A. amoebae* was reported as* Glossatella amoebae* [27, 55]. The first record of* A. amoebae* in Iraq was from the skin, buccal cavity, and gills of* C. idella* from Babylon Fish Farm [20]. So far this parasite has five fish host species in Iraq [17].

*Apiosoma cylindriformis* (Chen, 1955) was reported from gills of* C. idella* [20, 24, 27], buccal cavity and gills of* C. carpio* [27], and gills of* H. molitrix* [20, 21, 27]. Al-Zubaidy [27] reported* A. cylindriformis* as* Glossatella cylindriformis*. The first record of* A. cylindriformis* in Iraq was from gills of* C. idella* and* H. molitrix* from Babylon Fish Farm [20]. So far,* A. cylindriformis* has seven fish host species in Iraq [17].

*Apiosoma minuta* (Chen, 1961) Lom, 1966, was recorded from skin of* C. carpio* [45, 47]. This was its first record in Iraq. So far,* A. minuta* has two fish host species in Iraq as it was recently recorded from* Luciobarbus xanthopterus* by Al-Salmany [71].

*Apiosoma piscicola* Blanchard, 1885, was recorded from skin, buccal cavity, and gills of* C. idella* [27, 51], skin, buccal cavity, and gills of* C. carpio* [27, 29, 51, 55], and buccal cavity and gills of* H. molitrix* [20, 21, 27, 29, 51]. It is appropriate to mention here that* A. piscicola* was reported as* Glossatella piscicola* [27].* A. piscicola* was recorded for the first time in Iraq from skin, buccal cavity, and gills of* C. idella*,* C. carpio* and* H. molitrix* from Al-Suwaira and Al-Latifiya Fish Farms [51]. So far, this parasite has ten fish host species in Iraq [17].

*Apiosoma poteriformis* (Timofeev, 1962) Lom, 1966, was recorded from skin and gills of* C. idella* [20, 24, 27] and buccal cavity and gills of* C. carpio* [27]. It is appropriate to mention here that* A. poteriformis* was reported as* Glossatella poteriformis* by Al-Zubaidy [27].* A. poteriformis *was recorded for the first time in Iraq from gills of* C. idella* from Babylon Fish Farm [20]. So far, this parasite has three fish host species in Iraq [17].

*Trichodina cottidarum* Dogiel, 1948, was recorded from skin and gills of* C. carpio* [45, 47, 49, 54, 59, 60] and skin and gills of* H. molitrix* [45, 47].* T. cottidarum* was recorded for the first time in Iraq from gills of* C. carpio* from a manmade lake at Baghdad city [72]. So far, this parasite has 13 fish host species in Iraq [17].

*Trichodina domerguei* (Wallengren, 1897) was recorded from skin, fins, buccal cavity, and gills of* C. idella* [24, 27, 29, 43, 51, 56], skin, fins, buccal cavity, and gills of* C. carpio* [25, 27, 29, 30, 35, 43, 51, 53, 55, 56, 59], skin, buccal cavity, and gills of* H. molitrix* [20, 27, 51], and skin and gills of* L. abu* [23, 56]. The first record of* T. domerguei* in Iraq was from skin, fins, and gills of eight freshwater fish species from Tigris River, Al-Tharthar Lake, and fish markets in Baghdad city [73]. So far,* T. domerguei* has 39 host species in Iraq [17] and, therefore, it is the most prevalent ciliate species among fishes of Iraq.

*Trichodina gracilis* Polyanskii, 1955, was recorded from skin of* C. carpio* [45, 47]. This was its first record in Iraq. So far, it has three fish host species in Iraq [17].

*Trichodina nigra* Lom, 1960, was recorded from skin and gills of the three carp species:* C. idella* [29],* C. carpio* [27, 29, 35, 49, 55, 59, 60], and* H. molitrix* [27, 29]. The first record of* T. nigra* in Iraq was from skin and gills of* C. carpio* and gills of* H. molitrix* from Babylon Fish Farm [27]. So far,* T. nigra* has nine host species in Iraq [17].

Unidentified specimen of* Trichodina* was recorded from* L. abu* [50] with no mention to site of infection. In addition to 24 recognized* Trichodina* species so far recorded from fishes of Iraq, some unidentified species of* Trichodina* were so far recorded from six fish species [17].

*Tripartiella amurensis* (Chan, 1961) was recorded from skin of* C. carpio* [45, 47]. This was its first record in Iraq. No more records are so far known for* T. amurensis* in Iraq [17].

### 5.4. Phylum Myxozoa

The phylum Myxozoa is represented in farm fishes of Babylon province with five species: one species belonged to the genus* Myxobilatus* and four species to the genus* Myxobolus*. 

 Phylum Myxozoa

 Class Myxosporea
   Order Bivalvulida
     Family Sphaerosporidae
       
*Myxobilatus legeri* (Cépède, 1905)
     Family Myxobolidae
       
*Myxobolus dogieli* Bykhovskaya-Pavlovskaya & Bykhovski, 1940
       
*Myxobolus muelleri* Bütschli, 1882
       
*Myxobolus oviformis* Thélohan, 1892
       
*Myxobolus pfeifferi* Thélohan, 1895

*Myxobilatus legeri* (Cépède, 1905), erroneously reported as* Myxobllatus legerl*, with no given authority, description, and illustration, was recorded from skin and gills of* C. carpio* [54]. This was the only record of* M. legeri* in Iraq [17].

*Myxobolus dogieli* Bykhovskaya-Pavlovskaya & Bykhovski, 1940, was recorded from kidneys and gallbladder of* L. abu* [56]. The first record of* M. dogieli* in Iraq was mainly from the external surface of heart, liver, and ovaries of* L. abu* from Tigris River at Baiji town [74]. So far,* M. dogieli* has nine host species in Iraq [17].

*Myxobolus muelleri* Bütschli, 1882, was recorded from intestine and liver of* C. carpio* [27] with the specific name spelled as* mülleri*. The first record of* M. muelleri* in Iraq was from gills of* Luciobarbus xanthopterus*, reported as* B. xanthopterus* [70]. So far,* M. muelleri* has eight host species in Iraq [17].

*Myxobolus oviformis* Thélohan, 1892, was recorded from skin, intestine, and kidneys of* C. carpio* [27, 54, 59]. The first report of* M. oviformis* in Iraq was from gill arches and heart of four fish species [70]. So far,* M. oviformis* has 20 fish host species in Iraq [17].

*Myxobolus pfeifferi* Thélohan, 1895, was recorded from gills, intestine, liver, kidneys, and gallbladder of* C. idella* [29], gills, gallbladder, intestine, kidneys, and liver of* C. carpio* [25, 27, 29, 53, 59], gills, liver, and intestine of* H. molitrix* [27, 29], and gills, intestinal wall, and gonads of* L. abu* [23]. The first report of* M. pfeifferi* in Iraq was from gills of* Acanthobrama marmid* from Tigris River at Mosul city [75]. So far,* M. pfeifferi* is the prevalent myxozoan among fishes of Iraq as it has 35 fish host species [17].

### 5.5. Phylum Platyhelminthes: Class Trematoda

The class Trematoda of the phylum Platyhelminthes is represented in farm fishes of Babylon province with five species; two species belonged to the genera* Apharyngostrigea* and* Ascocotyle* and three species to the genus* Diplostomum*.

 Phylum Platyhelminthes

 Class Trematoda
   Order Diplostomida
     Family Strigeidae
       
*Apharyngostrigea cornu* (Zeder, 1800)
     Family Diplostomidae
       
*Diplostomum indistinctum* (Guberlet, 1923) Hughes, 1929
       
*Diplostomum paraspathaceum* Schigin, 1965
       
*Diplostomum spathaceum* (Rudolphi, 1819) Olsson, 1876
   Order Plagiorchiida
     Family Heterophyidae
       
*Ascocotyle coleostoma* (Looss, 1896) Looss, 1899

*Apharyngostrigea cornu* (Zeder, 1800) was recorded as metacercaria from mesentery, coelom and liver of* C. carpio* [52]. This was its first report in Iraq. No more records are so far known on the occurrence of* A. cornu* from fishes of Iraq [17]. The adult worm of this parasite was detected from the intestine of the purple heron* Ardea purpurea* in Bahr Al-Najaf Depression [76].

*Ascocotyle coleostoma* (Looss, 1896) Looss, 1899, was recorded as metacercaria from gills of* H. fossilis* [56]. This parasite was reported for the first time in Iraq from gills of* H. fossilis* and* L. abu* from Diyala River [77].* A. coleostoma* has so far 34 fish host species in Iraq [17]. The adult worm of* A. coleostoma* was detected from the grey heron* A. cinerea* in Babylon (now Al-Furat) Fish Farm [78].

*Diplostomum indistinctum* (Guberlet, 1923) Hughes, 1929, was recorded as metacercaria from eyes of* H. molitrix* [43]. The first occurrence of metacercariae of* D. indistinctum* was from eyes of* Luciobarbus esocinus*, reported as* B. esocinus* from fish market in Mosul city [79]. No more records are so far known on the occurrence of this parasite from fishes of Iraq [17].

*Diplostomum paraspathaceum* Schigin, 1965, was recorded as metacercaria from eyes of both* C. idella* [43] and* C. carpio* [43]. This was its first record in Iraq. No more hosts are so far known for this parasite in Iraq [17].

*Diplostomum spathaceum* (Rudolphi, 1819) Olsson, 1876, was recorded as metacercaria from eyes of the three carp species:* C. idella* [43, 56],* C. carpio* [29, 43], and* H. molitrix* [43, 56]. The first occurrence of metacercariae of* D. spathaceum* was from eyes of* C. luteus*, reported as* B. luteus*,* Cyprinion macrostomum*, and* C. carpio* from Dokan Lake [80]. Thirty-four hosts are so far known for this parasite in fishes of Iraq [17]. Metacercariae of* Diplostomum* spp. are responsible for worm cataract which causes fish blindness [81]. Adult worms of this parasite were found in the intestine of some fish-eating birds such as the silver gull* Larus argentatus* in Bahr Al-Najaf Depression [76].

### 5.6. Phylum Platyhelminthes: Class Monogenea

The class Monogenea of the phylum Platyhelminthes is represented in farm fishes of Babylon province with 45 species: 12 species of the genus* Gyrodactylus*, 26 species of* Dactylogyrus*, and one species of each of the genera* Pseudacolpenteron*,* Diplozoon*,* Eudiplozoon*,* Paradiplozoon*, and* Microcotyle* in addition to some unidentified species of* Dactylogyrus* and* Diplozoon*. It is appropriate to mention here that this group is considered as Monogenea by some electronic sites [9–12] but as Monogenoidea in some references [14, 66]. 

 Phylum Platyhelminthes

 Class Monogenea
   Order Gyrodactylidea
     Family Gyrodactylidae
       
*Gyrodactylus baicalensis* Bogolepova, 1950
       
*Gyrodactylus ctenopharngodontis* Ling in Gusev, 1952
       
*Gyrodactylus elegans* von Nordmann, 1832
       
*Gyrodactylus kherulensis* Ergens, 1974
       
*Gyrodactylus macracanthus* Hukuda, 1940
       
*Gyrodactylus malmbergi* Ergens, 1961
       
*Gyrodactylus markevitschi* Kulakovskaya, 1952
       
*Gyrodactylus medius* Kathariner, 1895
       
*Gyrodactylus menschikowi* Gvosdev, 1950
       
*Gyrodactylus salaris* Malmberg, 1957
       
*Gyrodactylus sprostonae* Ling, 1962
       
*Gyrodactylus vicinus* Bychowsky, 1957
   Order Dactylogyridea
     Family Dactylogyridae
       
*Dactylogyrus achmerowi* Gusev, 1955
       
*Dactylogyrus amurensis* Akhmerov, 1952
       
*Dactylogyrus anchoratus* (Dujardin, 1845) Wagener, 1857
       
*Dactylogyrus arcuatus* Yamaguti, 1942
       
*Dactylogyrus barbioides* Gusev, Ali, Abdul-Ameer, Amin & Molnár, 1993
       
*Dactylogyrus cornu* Linstow, 1878
       
*Dactylogyrus crassus* Kulwiec, 1927
       
*Dactylogyrus ctenopharyngodonis* Achmerow, 1952
       
*Dactylogyrus dogieli* Gusev, 1953
       
*Dactylogyrus ergensi* Molnár, 1964
       
*Dactylogyrus extensus* Mueller & Van Cleave, 1932
       
*Dactylogyrus gobii* Gvosdev, 1950
       
*Dactylogyrus hypophthalmichthys* Akhmerov, 1952
       
*Dactylogyrus inexpectatus* Izjumova, in Gusev, 1955
       
*Dactylogyrus jamansajensis* Osmanov, 1958
       
*Dactylogyrus lamellatus* Akhmerov, 1952
       
*Dactylogyrus latituba* Gusev, 1955
       
*Dactylogyrus lopuchinae* Jukhimenko, 1981
       
*Dactylogyrus minutus* Kulwiec, 1927
       
*Dactylogyrus navicularis* A. Gusev, 1955
       
*Dactylogyrus phoxini* Malevitskaia, 1949
       
*Dactylogyrus propinquus* Bychowsky, 1931
       
*Dactylogyrus sahuensis* Ling in Chen et al., 1973
       
*Dactylogyrus simplex* Bychowsky, 1936
       
*Dactylogyrus skrjabini* Akhmerov, 1954
       
*Dactylogyrus vastator* Nybelin, 1924
       
*Dactylogyrus* spp.
       
*Pseudacolpenteron pavlovskii* Bychowsky & Gussev, 1955
   Order Mazocraeidea
     Family Diplozoidae
       
*Diplozoon paradoxum* Nordmann, 1832
       
*Diplozoon* sp.
       
*Eudiplozoon nipponicum* (Goto, 1891)
       
*Paradiplozoon barbi* (Reichenbach-Klinke, 1951)
     Family Microcotylidae
       
*Microcotyle donavini* van Beneden & Hesse, 1863

*Gyrodactylus baicalensis* Bogolepova, 1950, was recorded from skin, fins, and gills of* C. carpio* [25, 27, 29, 52]. The first report of* G. baicalensis* in Iraq was from skin, buccal cavity, and gills of* C. carpio* from Al-Suwaira and Al-Latifiya Fish Farms [52]. So far,* G. baicalensis* has eight fish host species in Iraq [17].

*Gyrodactylus ctenopharngodontis* Ling in Gusev, 1952, was recorded from skin, fins, buccal cavity, and gills of* C. idella* [24, 27]. The first report of* G. ctenopharngodontis* in Iraq was from gills of* C. idella* from Babylon Fish Farm [24]. No more hosts are so far recorded for this parasite in Iraq [17].

*Gyrodactylus elegans* von Nordmann, 1832, was recorded from skin, fins, buccal cavity, and gills of both* C. idella* [27, 29, 52] and* C. carpio* [25–27, 29, 30, 32, 33, 43, 50, 52, 53, 55, 56, 60] as well as from gills of* H. molitrix* [29] and from* L. abu* [50] with no mention to site of infection. The first report of* G. elegans* in Iraq was from* C. carpio* from Al-Zaafaraniya Fish Farm and* L. abu* from Al-Latifiya Fish Farm [50]. So far,* G. elegans* has 23 fish host species in Iraq [17].

*Gyrodactylus kherulensis* Ergens, 1974, was recorded from skin and gills of* C. idella* [27] and skin, fins, and gills of* C. carpio* [18, 27]. The first report of* G. kherulensis* in Iraq was from gills of* C. carpio* from Babylon Fish Farm [18]. So far,* G. kherulensis* has four fish host species in Iraq [17].

*Gyrodactylus macracanthus* Hukuda, 1940 (reported as* G. paralatus* Gusev, 1955), was recorded from skin and gills of* C. carpio* [27] and skin, fins, and buccal cavity of* H. molitrix* [27]. This was its first report in Iraq. Later on, it was reported from both hosts [82] as* G. paralatus* also. According to Gussev [83] and Pugachev et al. [14],* G. paralatus* is a synonym of* G. macracanthus*. No more hosts are so far known for* G. macracanthus* or its synonym* G. paralatus* from fishes of Iraq [17].

*Gyrodactylus malmbergi* Ergens, 1961, was recorded from skin, fins, and gills of* C. carpio* [27] and from skin and gills of* H. molitrix* [27]. This was its first report in Iraq and no more hosts are so far known for* G. malmbergi* from fishes of Iraq [17].

*Gyrodactylus markevitschi* Kulakovskaya, 1952, was recorded from skin and gills of* C. carpio* [27, 45, 47, 60]. The first report of this parasite in Iraq was from gills of* Capoeta trutta* (reported as* Varicorhinus trutta*) from Tigris River at Baiji town [74]. So far,* G. markevitschi* has six host species in Iraq [17].

*Gyrodactylus medius* Kathariner, 1895, was recorded from skin and fins of* C. carpio* [27]. This was its first record in Iraq. The year of authority of this parasite was erroneously given as 1893 instead of 1895 by Al-Zubaidy [27] as well as by three other references according to Mhaisen and Abdul-Ameer [15]. Also the authorship of this parasite was given as Katheriner instead of Kathariner according to MonoDB [10]. Now, this parasite has three host species in Iraq [17].

*Gyrodactylus menschikowi* Gvosdev, 1950, was recorded from skin and gills of* C. carpio* [60]. The first report of this parasite in Iraq was from gills and skin of* C. carpio* and skin, fins, and gills of* L. abu* both from Hilla River [84]. So far, no more hosts for this parasite are known in Iraq [17].

*Gyrodactylus salaris* Malmberg, 1957, was recorded from skin and gills of* C. carpio* [27, 29]. The first report of this parasite in Iraq was from gills and skin of* C. carpio* from Al-Furat Fish Farm [27]. The year of authority of this parasite was reported as 1956 instead of 1957 by Al-Zubaidy [27] and Al-Jadoaa [29]. No more hosts for this parasite are so far known in Iraq [17].

*Gyrodactylus sprostonae* Ling, 1962, was recorded fromskin and fins of* C. carpio* [27]. This was its first report in Iraq. Now, this parasite has seven host species in Iraq [17].

*Gyrodactylus vicinus* Bychowsky, 1957, was recorded from skin, fins, and gills of* C. carpio* [27, 35]. The first report of this parasite in Iraq was from skin, fins, and gills of* C. carpio* from Al-Furat Fish Farm [27]. Now, it has three host species in Iraq [17].

Finally, the unidentified* Gyrodactylus* species reported from* C. carpio* [34, 38] were the same 11 species which had been recorded in Al-Zubaidy [27]. In Iraq, so far 15 fish host species were reported for some unspecified* Gyrodactylus* species [17].

*Dactylogyrus achmerowi* Gusev, 1955, was recorded from gills of* C. carpio* [19, 27, 29, 30, 32, 33, 36, 54, 55, 60]. The first report of* D. achmerowi* in Iraq was from gills of* C. carpio* from Al-Wahda Fish Hatchery at Al-Suwaira and Babylon Fish Farm [19]. Now, it has 11 host species in Iraq [17].

*Dactylogyrus amurensis* Akhmerov, 1952, was recorded from gills of* C. carpio* [55]. This was its first report in Iraq. However, neither description and measurements nor illustration was given. So far, this parasite has two host species in Iraq [17].

*Dactylogyrus anchoratus* (Dujardin, 1845) Wagener, 1857, was recorded from gills of* C. carpio* [45, 47]. The first report and description of* D. anchoratus* in Iraq were from gills of* C. carpio* from Tigris River at Al-Zaafaraniya [85, 86]. Now, it has seven fish host species in Iraq [17].

*Dactylogyrus arcuatus* Yamaguti, 1942, was recorded from gills of* C. idella* [29] and skin, buccal cavity, and gills of* C. carpio* [25, 27, 35, 36, 49, 52, 55, 59, 60]. The first report of* D. arcuatus* in Iraq was from skin, buccal cavity, and gills of* C. carpio* from Al-Suwaira and Al-Latifiya Fish Farms [52]. Now, it has seven fish host species in Iraq [17].

*Dactylogyrus barbioides* Gusev, Ali, Abdul-Ameer, Amin & Molnár, 1993, was recorded from gills of* C. carpio* [60].* D. barbioides* was described as a new species from gills of* Barbus grypus* from Tigris River near Baiji town [87]. Now, it has three fish host species in Iraq [17].

*Dactylogyrus cornu* Linstow, 1878, was recorded from gills of* C. carpio* [27, 59].* D. cornu* was recorded for the first time in Iraq from gills of five fish species from Diyala River [88]. So far, it has 13 fish host species in Iraq [17].

*Dactylogyrus crassus* Kulwiec, 1927, was recorded from gills of* C. carpio* [45, 47, 55, 60].* D. crassus* was recorded for the first time in Iraq from gills of* C. carpio* from Al-Shark Al-Awsat Fish Farm [45]. It is appropriate to mention here that the specific name* crassus* was misspelled as* carassus* by Al-Rubaie et al. [55]. No more hosts are so far known for this parasite in Iraq [17].

*Dactylogyrus ctenopharyngodonis* Achmerow, 1952, was recorded from gills of* C. idella* from Al-Shark Al-Awsat Fish Farm [43]. This is the only report on the occurrence of* D. ctenopharyngodonis* in fishes of Iraq [17].

*Dactylogyrus dogieli* Gusev, 1953, was recorded from gills of* C. carpio* in fish cages and an earthen pond in Sadat Al-Hindiya [60]. The first report of* D. dogieli* was from five fish species from Euphrates River at Al-Musaib city [89] and its full description and illustration were published later by Al-Sa'adi et al. [90]. Six host species are so far known for* D. dogieli* in Iraq [17].

*Dactylogyrus ergensi* Molnár, 1964, was recorded from gills of* C. carpio* from Al-Furat Fish Farm [27]. This was its first report in Iraq. No more hosts were reported for* D. ergensi* in Iraq [17].

*Dactylogyrus extensus* Mueller & Van Cleave, 1932, was recorded from gills of* C. idella* [29], buccal cavity and gills of* C. carpio* [25, 27, 29, 30, 32, 33, 36, 43, 45, 47, 52, 54, 55, 59, 60], and gills of* H. molitrix* [29, 45, 47]. The first report of* D. extensus* in Iraq was from the buccal cavity and gills of* C. carpio* from Al-Suwaira and Al-Latifiya Fish Farms [52].* D. solidus* which was also recorded from the same host by Salih et al. [52] as well as by Mhaisen & Abul-Eis [25] and Al-Rubaie et al. [55] is considered as a synonym of* D. extensus* according to Gibson et al. [13].* D. extensus* and its synonym* D. solidus* have so far 17 fish host species in Iraq [17].

*Dactylogyrus gobii* Gvosdev, 1950, was recorded from skin and gills of* C. carpio* [45, 47, 60].* D. gobii* was recorded for the first time in Iraq from gills of* C. carpio* from Al-Shark Al-Awsat Fish Farm [45]. Now, it has three host species in Iraq [17].

*Dactylogyrus hypophthalmichthys* Akhmerov, 1952, was recorded from skin, buccal cavity, and gills of* H. molitrix* [21, 27, 43, 45, 47, 52, 56]. It is reliable to state here that* D. hypophthalmichthys* was reported as* Neodactylogyrus hypophthalmichthys* by Asmar et al. [56]. The first report of* D. hypophthalmichthys* in Iraq was from the buccal cavity and gills of* H. molitrix* from Al-Suwaira and Al-Latifiya Fish Farms [52].* H. molitrix* is the only host so far known for* D. hypophthalmichthys* in Iraq [17].

*Dactylogyrus inexpectatus* Izjumova, in Gusev, 1955, was recorded from skin and gills of* C. idella* [27, 29, 52], gills of* C. carpio* [27, 29], and gills of* H. molitrix* [29]. The first report of* D. inexpectatus* in Iraq was from skin and gills of* C. idella* from Al-Suwaira and Al-Latifiya Fish Farms [52]. Now, it has five host species in Iraq [17].

*Dactylogyrus jamansajensis* Osmanov, 1958, was recorded from gills of* C. carpio* [45, 47]. The first report of* D. jamansajensis* in Iraq was from gills of* C. luteus* from manmade lakes, north of Baghdad [91]. Now, it has five host species in Iraq [17].

*Dactylogyrus lamellatus* Akhmerov, 1952, was recorded from skin, fins, buccal cavity, and gills of* C. idella* [24, 27, 43, 52, 56] and gills of* C. carpio* [27]. The first report of* D. lamellatus* in Iraq was from the skin, buccal cavity, and gills of* C. idella* from Al-Suwaira and Al-Latifiya Fish Farms [52]. Now, it has three host species in Iraq [17].

*Dactylogyrus latituba* Gusev, 1955, was recorded from gills of both* C. idella* [27] and* C. carpio* [25, 27] as well as from the buccal cavity and gills of* H. molitrix* [27]. The first report of* D. latituba* in Iraq was from gills of* C. luteus* from manmade lakes, north of Baghdad [91]. Now, it has four host species in Iraq [17].

*Dactylogyrus lopuchinae* Jukhimenko, 1981, was recorded from gills of* C. carpio* [45, 47, 54].* D. lopuchinae* was recorded for the first time in Iraq from gills of* C. carpio* from Al-Shark Al-Awsat Fish Farm [45]. No more hosts are so far known for this parasite in Iraq [17].

*Dactylogyrus minutus* Kulwiec, 1927, was recorded from skin, fins, and gills of* C. carpio* [27, 30, 32, 33, 45, 47, 55, 56, 59, 60]. The first report on this parasite in Iraq was from gills of* C. carpio* from Tigris River at Al-Zaafaraniya, south of Baghdad, and Al-Qadisia Dam Lake [85], while its description and illustration were given later by Mhaisen et al. [86]. So far,* D. minutus* has 12 fish species in Iraq [17].

*Dactylogyrus navicularis* A. Gusev, 1955, was recorded from fins, buccal cavity, and gills of* C. carpio* [27, 35, 60]. The first report of* D. navicularis* in Iraq was from the buccal cavity, fins, and gills of* C. carpio* from Al-Furat Fish Farm [27].* C. carpio* is the only host so far known for* D. navicularis* in Iraq [17].

*Dactylogyrus phoxini* Malevitskaia, 1949, was recorded from skin and gills of* C. carpio* [45, 47, 60]. The first record of* D. phoxini* in Iraq was in June 1995 from gills of* C. carpio* from Tigris River at Al-Zaafaraniya but the report was published later by Balasem et al. [92]. This parasite has so far only two host species in Iraq [17].

*Dactylogyrus propinquus* Bychowsky, 1931, was recorded from gills of* C. carpio* [27, 35, 49]. The first report of* D. propinquus* in Iraq was from gills of* C. carpio* from Al-Furat Fish Farm [27].* C. carpio* is the only host so far known for* D. propinquus* in Iraq [17].

*Dactylogyrus sahuensis* Ling in Chen et al., 1973, was recorded from fins and gills of* C. carpio* [27]. The first report of* D. sahuensis* in Iraq was from fins and gills of* C. carpio* from Al-Furat Fish Farm [27].* C. carpio* is the only host so far known for* D. sahuensis* in Iraq [17].

*Dactylogyrus simplex* Bychowsky, 1936, was recorded from* C. carpio* [49] with no mention to site of infection. The first report of* D. simplex* in Iraq was from gills of* C. carpio* from the new fish farm of the Fish Research Center at Al-Zaafaraniya [93].* D. simplex* has so far three host species in Iraq [17].

*Dactylogyrus skrjabini* Akhmerov, 1954, was recorded from buccal cavity and gills of* C. carpio* [27, 45, 47] and buccal cavity and gills of* H. molitrix* [21, 27, 52]. The first report of* D. skrjabini* in Iraq was from buccal cavity and gills of* H. molitrix* from Al-Suwaira and Al-Latifiya Fish Farms [52]. Now,* D. skrjabini* has six host species in Iraq [17].

*Dactylogyrus vastator* Nybelin, 1924, was recorded from gills of* C. idella* [27] and skin and gills of* C. carpio* [26, 27, 30, 32, 33, 35, 36, 43, 49, 52, 53, 56, 59, 60, 64]. The first report of* D. vastator* from Iraq was from skin and gills of* C. macrostomum* from Tigris River at Baghdad [94]. So far,* D. vastator* was reported from 33 fish host species from north, middle, and south of Iraq [17].

Unidentified* Dactylogyrus* species were recorded from skin and gills of* C. idella* [56] and from skin, buccal cavity, and gills of* C. carpio* [34, 38, 56]. Some of these specimens were larval stages [56], while the unidentified* Dactylogyrus* species of Al-Zubaidy et al. [34, 38] were the same 15 species which were recorded in Al-Zubaidy [27]. In Iraq, so far nine fish host species were reported for some unspecified* Dactylogyrus* species [17].

*Pseudacolpenteron pavlovskii* Bychowsky & Gussev, 1955, was recorded from fins and gills of* C. carpio* [25, 27, 45, 47] and gills of* H. molitrix* [27]. The first report of* P. pavlovskii* from Iraq was from skin and gills of* C. carpio *from Babylon Fish Farm [25]. This species has so far only two host species in Iraq [17].

*Diplozoon paradoxum* Nordmann, 1832, was recorded from gills of* C. carpio* [60]. This parasite was reported for the first time in Iraq from gills of* Carasobarbus luteus*, reported as* Barbus luteus*, from Al-Husainia creek, Karbala province [95]. Now, it has five fish hosts in Iraq [17].

Unidentified* Diplozoon* species were recorded from gills of* C. carpio* [43]. Some other unidentified* Diplozoon* species occurred as larvae in 12 fish host species in Iraq [17].

*Eudiplozoon nipponicum* (Goto, 1891) was recorded from gills of* C. carpio* [60]. This parasite was recorded for the first time in Iraq from gills of* C. carpio* from a manmade lake in Baghdad [96] as* Diplozoon nipponicum* but then it was reported by its valid name* E. nipponicum* by all subsequent researchers. So far, three host species are known for* E. nipponicum* in Iraq [17].

*Paradiplozoon barbi* (Reichenbach-Klinke, 1951) was recorded from gills of* C. carpio* [27] as* Diplozoon barbi*. This parasite was reported for the first time in Iraq from gills of* Chondrostoma nasus*,* C. regium*, and* C. carpio* from Tigris River at Baghdad  [97] as* Diplozoon barbi*. Also, all the subsequent records in the Iraqi literature, except the checklists of Mhaisen and Abdul-Ameer [16], referred to this parasite as* D. barbi*. According to Khotenovsky [98],* D. barbi* is a synonym of* P. barbi*. Eight host species are so far known for this parasite in Iraq [17].

*Microcotyle donavini* van Beneden & Hesse, 1863, was recorded from gills of* L. abu* from Babylon Fish Farm [22]. This was its first report in Iraq. Ten host species are so far known for* M. donavini* in Iraq [17].

### 5.7. Phylum Platyhelminthes: Class Cestoda

The class Cestoda of the phylum Platyhelminthes is represented in farm fishes of Babylon province with five species: one species of each of the genera* Bothriocephalus*,* Ligula*, and* Neogryporhynchus* as well as two species of* Proteocephalus*. 

 Phylum Platyhelminthes

 Class Cestoda
   Order Bothriocephalidea
     Family Bothriocephalidae
       
*Bothriocephalus acheilognathi* Yamaguti, 1934
   Order Diphyllobothriidea
     Family Diphyllobothriidae
       
*Ligula intestinalis* (L., 1758) Bloch, 1782
   Order Proteocephalidea
     Family Proteocephalidae
       
*Proteocephalus osculatus* (Goeze, 1782) Nybelin, 1942
       
*Proteocephalus torulosus* (Batsch, 1786) Nufer, 1905
   Order Cyclophyllidea
     Family Dilepididae
       
*Neogryporhynchus cheilancristrotus* (Wedl, 1855) Baer & Bona, 1960

*Bothriocephalus acheilognathi* Yamaguti, 1934, was recorded from the intestine of both* C. idella* [24, 56] and* C. carpio* [25–27, 52, 53, 62]. It is appropriate to mention here that this worm was reported by its synonym* B. opsariichthydis* by Salih et al. [52] and Al-Zubaidy [27]. The first report of* B. acheilognathi* in Iraq was from the intestine of* C. carpio* from different fish farms near Baghdad [99]. Two other species of* Bothriocephalus*,* B. gowkongensis* Yeh, 1955, and* B. opsariichthydis* Yamaguti, 1934, were also reported from Iraq [17]. According to Molnár [100], both these two species are considered as synonyms of* B. acheilognathi*. At the present time,* B. acheilognathi* and both of its above-named synonyms has so far a total of 21 host species in Iraq [17].

*Ligula intestinalis* (L., 1758) Bloch, 1782, was recorded from the body cavity of both* C. idella* [24, 27, 29] and* C. carpio* [27].* L. intestinalis* was reported for the first time in Iraq as a plerocercoid from the body cavity of* Leuciscus vorax* (reported as* A. vorax*) from Shatt Al-Arab River [101]. So far, this species has 13 fish host species in Iraq [17]. In Iraq, the adult stage of* L. intestinalis* was reported from the intestine of the moorhen* Gallinula chloropus chloropus* from around Baghdad [102].

*Proteocephalus osculatus* (Goeze, 1782) Nybelin, 1942, was recorded from the intestine of* C. carpio* [27]. The first report of this parasite in Iraq was from the intestine of* L. vorax* (reported as* A. vorax*) from Al-Tharthar Lake [103]. So far, this species has eight fish host species in Iraq [17].

*Proteocephalus torulosus* (Batsch, 1786) Nufer, 1905, was recorded from intestine of* C. carpio* [27]. The first report of this parasite in Iraq was from the intestine of* C. carpio* from a fish farm near Baghdad city [99]. So far, this species has two fish host species in Iraq [17].

*Neogryporhynchus cheilancristrotus* (Wedl, 1855) Baer & Bona, 1960, was recorded from the intestine of* C. carpio* by Al-Zubaidy [27] as* Gryporhynchus cheilancristrotus*. The first report of* N. cheilancristrotus* in Iraq was from the intestine of* L. abu* from Diyala River [104]. So far, this species has four fish host species in Iraq [17].

### 5.8. Phylum Nematoda

The phylum Nematoda is represented in farm fishes of Babylon province with three species: unidentified larval species of the genus* Contracaecum* as well as one species of each of the genera* Cucullanus* and* Rhabdochona*.

 Phylum Nematoda

 Class Secernentea
   Order Ascaridida
     Family Anisakidae
       
*Contracaecum* spp.
     Family Cucullanidae
       
*Cucullanus cyprini* Yamaguti, 1941
   Order Spirurida
     Family Rhabdochonidae
       
*Rhabdochona hellichi* (Srámek, 1901)

Unidentified larval species of* Contracaecum* was recorded from the intestinal wall, body cavity, liver, spleen, heart, and gonads of* C. carpio* [27, 29] and intestinal wall of* L. abu* [22, 37, 42]. The first report of* Contracaecum* spp. larvae in Iraq was from the body cavity and different viscera of 10 fish species from different inland waters of Iraq [70].* Contracaecum* spp. larvae have so far 40 fish host species in Iraq [17]. Adult worms of* Contracaecum* spp. were detected from six species of aquatic birds in Iraq,* Egretta alba*,* E. garzetta*,* Ardeola ralloides*,* Botaurus stellaris*,* Ardea purpurea*, and* Ceryle rudis*, from Bahr Al-Najaf Depression [76].

*Cucullanus cyprini* Yamaguti, 1941, was recorded from the intestine of* C. carpio* [27]. The first report of this parasite in Iraq was from the intestine of* Alburnus caeruleus* and* Luciobarbus xanthopterus* (reported as* B. xanthopterus*) from Al-Tharthar Lake [103]. So far, this species has 15 fish host species in Iraq [17].

*Rhabdochona hellichi* (Srámek, 1901), erroneously reported as* R. bellichi*, was recorded from intestine of the three carp species:* C. idella* [29],* C. carpio* [29], and* H. molitrix* [29]. Ali et al. [105] reported this parasite (also erroneously as* R. bellichi*) from the intestine and coelom of* L. xanthopterus* (reported as* B. xanthopterus*),* H. fossilis*, and* Mystus pelusius* (reported as* M. halepensis*). Eight fish species are so far known for this parasite in Iraq [17].

### 5.9. Phylum Acanthocephala

The phylum Acanthocephala is represented in farm fishes of Babylon province with two valid species of the genus* Neoechinorhynchus*. 

 Phylum Acanthocephala

 Class Eoacanthocephala
   Order Neoechinorhynchida
     Family Neoechinorhynchidae
       
*Neoechinorhynchus iraqensis* Amin, Al-Sady, Mhaisen & Bassat, 2001
       
*Neoechinorhynchus rutili* (Müller, 1780) Hamann, 1892

*Neoechinorhynchus iraqensis* Amin, Al-Sady, Mhaisen & Bassat, 2001, was recorded from intestine of both* C. carpio* [27] and* L. abu* [22, 50]. It is appropriate to mention here that this species was reported as* N. agilis* from* C. carpio* and* L. abu* by Al-Zubaidy [27] and Ali et al. [22], respectively.* N. agilis* is a misidentification of* N. iraqensis* [106]. The first report of* N. iraqensis* was as* species de novo* from the intestine of* L. abu* from the Euphrates River at Al-Fallujah region [107], while its description was given later by Amin et al. [108].* N. iraqensis* and the misidentified* N. agilis* have so far 24 fish host species in Iraq [17].

*Neoechinorhynchus rutili* (Müller, 1780) Hamann, 1892, was recorded from the intestine of the three carp species:* C. idella* [29],* C. carpio* [27, 29], and* H. molitrix* [29].* N. rutili* was firstly recorded by Herzog [70] from* L. xanthopterus* (reported as* B. xanthopterus*) from Tigris and Diyala rivers near Baghdad and from* L. abu* (reported as* Mugil abu*) from Citscher Oasis near Al-Fallujah.* N. rutili* has so far 16 fish host species in Iraq [17].

### 5.10. Phylum Arthropoda: Subphylum Crustacea

The subphylum Crustacea of the phylum Arthropoda is represented in farm fishes of Babylon province with nine species: one species of each of the genera* Argulus*,* Dermoergasilus*,* Paraergasilus*,* Lamproglena*, and* Lernaea* and three species of* Ergasilus* in addition to unidentified species of* Ergasilus*.  

 Phylum Arthropoda

 Subphylum Crustacea
   Class Maxillopoda
     Order Arguloida
       Family Argulidae
       
* Argulus foliaceus* (L., 1758)
     Order Cyclopoida
       Family Ergasilidae
       
* Dermoergasilus varicoleus* Ho, Jayarajan & Radhakrishnan, 1992
       
* Ergasilus barbi* Rahemo, 1982
       
* Ergasilus mosulensis* Rahemo, 1982
       
* Ergasilus sieboldi* von Nordmann, 1832
       
* Ergasilus* sp.
       
* Paraergasilus inflatus* Ho, Khamees & Mhaisen, 1996
       Family Lernaeidae
       
*Lamproglena pulchella* von Nordmann, 1832
       
* Lernaea cyprinacea* L., 1758

*Argulus foliaceus* (L., 1758) was recorded from gills of the three carp species:* C. idella* [27],* C. carpio* [25, 27], and* H. molitrix* [27] as well as from fins of* C. auratus* [58].* A. foliaceus* was reported for the first time in Iraq [70] from skin of* C. carpio* from Al-Zaafaraniya Fish-Culture Station and* C. luteus* (reported as* B. luteus*) from Al-Habbaniyah Lake [70].* A. foliaceus* has so far 16 fish host species in Iraq [17].

*Dermoergasilus varicoleus* Ho, Jayarajan & Radhakrishnan, 1992, was recorded from gills of* L. abu* [31, 40]. This crustacean was reported for the first time in Iraq from gills of* L. abu* from Shatt Al-Arab River [109].* D. varicoleus* has so far nine fish host species in Iraq [17].

*Ergasilus barbi* Rahemo, 1982, was recorded from gills of* L. abu* [46]. This crustacean was firstly detected from gills of* B. grypus* from Tigris River at Mosul city by Fattohy [75] and its full description as a new species was achieved by Rahemo [110].* E. barbi* has so far 13 fish host species in Iraq [17].

*Ergasilus mosulensis* Rahemo, 1982, was recorded from gills of the three carp species,* C. idella* [27],* C. carpio* [27], and* H. molitrix* [27] as well as gills of* L. abu* [46]. This crustacean was firstly detected from gills of* L. abu* from Tigris River at Mosul city by Fattohy [75] and its full description as a new species was achieved by Rahemo [110].* E. mosulensis* has so far 23 fish host species in Iraq [17].

*Ergasilus sieboldi* von Nordmann, 1832, was recorded from gills, buccal cavity, and skin of the three carp species:* C. idella* [27, 29],* C. carpio* [25, 27, 29, 51, 55], and* H. molitrix* [27, 29, 56]. The first report of* E. sieboldi* in Iraq was from gills of* L. vorax* (reported as* A. vorax*) from Al-Habbaniyah Lake [70].* E. sieboldi* has so far 26 fish host species in Iraq [17].

*Ergasilus* sp. was recorded from* L. abu* [50] with no mention to site of infection. In addition to 11 species of* Ergasilus* so far recorded from fishes of Iraq, some specimens of unidentified* Ergasilus* species were also reported from 12 fish species in Iraq [17].

*Paraergasilus inflatus* Ho, Khamees & Mhaisen, 1996, was recorded from gills of* H. molitrix* [27]. This crustacean was reported as a new species from gill rakers of* L. abu* from Shatt Al-Arab River [111].* D. varicoleus* has so far seven fish host species in Iraq [17].

*Lamproglena pulchella* von Nordmann, 1832, was recorded from gills of* C. carpio* [27]. The first report of* L. pulchella* in Iraq was from gills of* Chondrostoma regium* and* Capoeta trutta *(reported as* Varicorhinus trutta*) from Tigris River at Mosul city [112].* L. pulchella* has so far 19 fish host species in Iraq [17].

*Lernaea cyprinacea* L., 1758, was recorded from skin, fins, and gills of the three carp species,* C. idella* [24, 27–29, 43, 51],* C. carpio* [6, 25–31, 39, 41, 43, 44, 48, 51, 53–55, 57, 61, 63], and* H. molitrix* [21, 27–29, 43], as well as from gills of* L. abu* [23]. The first report of the anchor worm* L. cyprinacea* in Iraq was from skin, fins, buccal cavity, pharyngeal cavity, gills, and anus of seven freshwater fish species from Al-Zaafaraniya Fish-Culture Station [113].* L. cyprinacea* is the commonest crustacean among fishes of Iraq as it has so far 30 fish host species in Iraq [17].

### 5.11. Phylum Mollusca

The phylum Mollusca is represented in farm fishes of Babylon province with only one parasite species of the genus* Unio*. The systematic account of this parasite, followed by parasite-host list, is given here. 

 Phylum Mollusca

 Class Bivalvia
   Order Unionoida
     Family Unionidae
       
*Unio pictorum* (Linnaeus, 1758)

*Unio pictorum* (Linnaeus, 1758) was recorded from gills of both* C. carpio* [27, 29] and* H. molitrix* [27]. The first report of the glochidial larvae of* U. pictorum* in Iraq was from gills of eight freshwater fish species from Diyala River [104]. It is appropriate to mention here that the authority of* U. pictorum* was erroneously stated as Zhadin, 1938, in all the Iraqi literature except Al-Salmany [71].* U. pictorum* has so far 24 fish host species in Iraq [17].

## 6. Host-Parasite List

The following host-parasite list for fish parasites in fish farms of Babylon province is compiled. For each host, the scientific names of all recorded parasites are alphabetically enlisted under their major parasitic groups. To economize space, references of previous records for each parasite species are not given here. These can be obtained from the account of each concerned parasite species in the part of major groups of parasitic fauna within the results and discussion part.

### 6.1. *Cyprinus carpio* Linnaeus, 1758

 Mastigophora:* Ichthyobodo necator*.
 Apicomplexa:* Eimeria dogieli*,* E. mylopharyngodoni*, and* Haemogregarina* sp.
 Ciliophora:* Apiosoma amoebae*,* A. cylindriformis*,* A. minuta*,* A. piscicola*,* A. poteriformis*,* Chilodonella cyprini*,* Ichthyophthirius multifiliis*,* Trichodina cottidarum*,* T. domerguei*,* T. gracilis*,* T. nigra*, and* Tripartiella amurensis*.
 Myxozoa:* Myxobilatus legeri*,* Myxobolus muelleri*,* M. oviformis*, and* M. pfeifferi*.
 Trematoda:* Apharyngostrigea cornu*,* Diplostomum paraspathaceum*, and* D. spathaceum*.
 Monogenea:* Dactylogyrus achmerowi*,* D. amurensis*,* D. anchoratus*,* D. arcuatus*,* D. barbioides*,* D. cornu*,* D. crassus*,* D. dogieli*,* D. ergensi*,* D. extensus*,* D. gobii*,* D. inexpectatus*,* D. jamansajensis*,* D. lamellatus*,* D. latituba*,* D. lopuchinae*,* D. minutus*,* D. navicularis*,* D. phoxini*,* D. propinquus*,* D. sahuensis*,* D. simplex*,* D. skrjabini*,* D. vastator*,* Dactylogyrus* spp.,* Diplozoon paradoxum*,* Diplozoon *sp.,* Eudiplozoon nipponicum*,* Gyrodactylus baicalensis*,* G. elegans*,* G. kherulensis*,* G. macracanthus*,* G. malmbergi*,* G. markewitschi*,* G. medius*,* G. menschikowi*,* G. salaris*,* G. sprostonae*,* G. vicinus*,* Paradiplozoon barbi*, and* Pseudacolpenteron pavlovskii*.
 Cestoda:* Bothriocephalus acheilognathi*,* Ligula intestinalis*,* Neogryporhynchus cheilancristrotus*,* Proteocephalus osculatus*, and* P. torulosus*.
 Nematoda:* Contracaecum* sp.,* Cucullanus cyprini*, and* Rhabdochona hellichi*.
 Acanthocephala:* Neoechinorhynchus iraqensis* and* N. rutili*.
 Crustacea:* Argulus foliaceus*,* Ergasilus mosulensis*,* E. sieboldi*,* Lamproglena pulchella*, and* Lernaea cyprinacea*.
 Mollusca:* Unio pictorum*.

### 6.2. *Ctenopharyngodon idella* (Valenciennes, 1844)

 Ciliophora:* Apiosoma amoebae*,* A. cylindriformis*,* A. piscicola*,* A. poteriformis*,* Chilodonella cyprini*,* Ichthyophthirius multifiliis*,* Trichodina domerguei*, and* T. nigra*.
 Myxozoa:* Myxobolus pfeifferi*.
 Trematoda:* Diplostomum paraspathaceum* and* D. spathaceum*.
 Monogenea:* Dactylogyrus arcuatus*,* D. ctenopharyngodonis*,* D. extensus*,* D. inexpectatus*,* D. lamellatus*,* D. latituba*,* D. vastator*,* Dactylogyrus* sp.,* Gyrodactylus ctenopharyngodontis*,* G. elegans*, and* G. kherulensis*.
 Cestoda:* Bothriocephalus acheilognathi* and* Ligula intestinalis*.
 Nematoda:* Rhabdochona hellichi*.
 Acanthocephala:* Neoechinorhynchus rutili*.
 Crustacea:* Argulus foliaceus*,* Ergasilus mosulensis*,* E. sieboldi*, and* Lernaea cyprinacea*.

### 6.3. *Hypophthalmichthys molitrix* (Valenciennes, 1844)

 Ciliophora:* Apiosoma amoebae*,* A. cylindriformis*,* A. piscicola*,* Chilodonella cyprini*,* Ichthyophthirius multifiliis*,* Trichodina cottidarum*,* T. domerguei*, and* T. nigra*.
 Myxozoa:* Myxobolus pfeifferi*.
 Trematoda:* Diplostomum indistinctum* and* D. spathaceum*.
 Monogenea:* Dactylogyrus extensus*,* D. hypophthalmichthys*,* D. inexpectatus*,* D. latituba*,* D. skrjabini*,* Gyrodactylus elegans*,* G. macracanthus*,* G. malmbergi*, and* Pseudacolpenteron pavlovskii*.
 Nematoda:* Rhabdochona hellichi*.
 Acanthocephala:* Neoechinorhynchus rutili*.
 Crustacea:* Argulus foliaceus*,* Ergasilus mosulensis*,* E. sieboldi*,* Lernaea cyprinacea*, and* Paraergasilus inflatus*.
 Mollusca:* Unio pictorum*.

### 6.4. *Carassius auratus* (Linnaeus, 1758)

 Crustacea:* Argulus foliaceus*.

### 6.5. *Heteropneustes fossilis* (Bloch, 1794)

 Trematoda:* Ascocotyle coleostoma*.

### 6.6. *Liza abu* (Heckel, 1843)

 Ciliophora:* Trichodina domerguei* and* Trichodina* sp.
 Myxozoa:* Myxobolus dogieli* and* M. pfeifferi*.
 Monogenea:* Gyrodactylus elegans* and* Microcotyle donavini*.
 Nematoda:* Contracaecum* sp.
 Acanthocephala:* Neoechinorhynchus iraqensis*.
 Crustacea:* Dermoergasilus varicoleus*,* Ergasilus barbi*,* E. mosulensis*,* Ergasilus* sp., and* Lernaea cyprinacea*.
